# An Opportunity to Harmonise the Approach to Patients' Care Pathways for Rare and Complex Diseases: RarERN Path™

**DOI:** 10.3389/frhs.2022.935014

**Published:** 2022-07-13

**Authors:** Rosaria Talarico, Diana Marinello, Sara Cannizzo, Ilaria Palla, Simone Ticciati, Andrea Gaglioti, Andrzej Rys, Carlo Milli, Domenica Taruscio, Marta Mosca, Giuseppe Turchetti

**Affiliations:** ^1^Rheumatology Unit, Department of Internal Medicine, Azienda Ospedaliero Universitaria Pisana, Pisa, Italy; ^2^Institute of Management, Scuola Superiore Sant'Anna, Pisa, Italy; ^3^DG Sante, Brussels, Belgium; ^4^Administrative Unit Chief, Azienda Ospedaliero Universitaria Pisana, Pisa, Italy; ^5^National Centre for Rare Diseases, Istituto Superiore di Sanità, Rome, Italy; ^6^Rheumatology Unit, University of Pisa, Pisa, Italy

**Keywords:** rare diseases, organisation of care, patients' care pathways, healthcare system, RarERN Path™

## Abstract

As a matter of fact, organisation always matters when discussing about healthcare, since it is fundamental in order to ensure the delivery of the most appropriate care to patients in the most appropriate way. Unfortunately, the pandemic brought by the severe acute respiratory syndrome-coronavirus 2 (SARS-CoV-2) imposed a huge reorganisation of the healthcare systems, with several repercussions on the care of several chronic conditions, that were in many cases discontinued. This was the case of rare diseases (RDs), conditions that even under normal circumstances can experience diagnostic delays and difficulties in receiving appropriate care. The context of the European Reference Networks (ERNs) represents one of the most appropriate settings for the creation of organisational reference models for patient care pathways (PCP). As a matter of fact, the main mission of ERNs is to improve the care of patients with RDs in Europe through a patient-centred approach, thanks to real multistakeholder involvement. For this reason, in the last years, an extensive effort has been made towards the creation of a methodological approach aimed at providing organisational reference models for PCP in RDs across the different Member States. In fact, in order to develop the reference model, a structured methodology was created to enable the design of the PCP based on a deep sharing of expertise on high-quality care and characterised by a strong patient-centred approach: RarERN Path™. Among the different stakeholders that need to be involved in planning strategic actions to ensure care also during an emergency, patients' representatives, healthcare professionals, hospital managers, and experts in healthcare organisations play a crucial role.

## Introduction

Rare diseases (RDs) affect more than 30 million people in Europe and many of them still have limited access to timely diagnosis and high-quality treatment. Moreover, improving the scientific evidence in RDs can often represent a challenge due to the low number of patients. In order to address these challenges, the European Commission launched the European Reference Networks (ERNs), virtual networks involving healthcare providers (HCPs) across the European Union (EU). The mission of the ERNs is to tackle low prevalence and RDs that require highly specialised treatment and a concentration of knowledge and resources and therefore to promote equity of care (1).

It is well known that no health care system model can be considered the most appropriate and extensively accepted from different points of view. Even within the same country, several dissimilarities can be often detected, and they can be related to different aspects, including structures, resources, and other specific characteristics of the health care system itself. However, there is a common aspect that each health system requires, and it is represented by an appropriate level of organisation. As a matter of fact, an organisation always matters when discussing healthcare, since it is fundamental in order to ensure the delivery of the most appropriate care to patients in the most appropriate way (2, 3).

In the last years, the severe acute respiratory syndrome-coronavirus 2 (SARS-CoV-2) pandemic imposed a huge reorganisation of the healthcare systems, due to the care needed for patients with COVID-19 and a consequent concentration of resources in the COVID-19 units^1^. The main repercussion of this necessary reorganisation was represented by the concentration of human resources from different units to be often entirely devoted to COVID-19 units. This resulted in many units being often closed or reduced to the provision of emergency services. Unfortunately, due to these big challenges, the care of several chronic conditions was in many cases discontinued and patients and healthcare professionals treating these conditions had to cope with a new arduous scenario. This was the case with RDs (4). Even under normal circumstances, patients with RDs can experience diagnostic delays, and difficulties in receiving appropriate support and care, and this may have a high impact on prognosis as well as morbidity and mortality. During an emergency, vulnerable patients are even more vulnerable.

Therefore, the relevant impact that COVID-19 had during the different waves and is still having on the provision of services to chronic patients highlights the need to develop specific organisational strategies for healthcare systems. Every single challenge that the health systems are experiencing in these hard times highlights how organisation matters, especially during a health emergency (5). In fact, the only way to ensure appropriate care to chronic disease patients during an emergency is to have detailed strategic plans for health care systems; for this purpose, it is desirable that specific actions may create or optimise existing organisational models for the care of chronic diseases. This is particularly crucial not only in case of future emergencies but also in other situations that might threaten the provision of routine care. These pathways should be based on efficient healthcare planning and referral systems that would help better define the different appropriate tasks of the professionals involved in patient care (6). Emergency plans should be designed or adapted to ensure that the diagnostic, monitoring, and therapeutic pathways of rare disease patients remain accessible for these patients. To do that, detailed organisational procedures need to be defined as soon as possible, identifying the different healthcare services to be maintained and preserved in case of a new pandemic or other health emergencies. Thus, it is desirable that *ad hoc* organisational models are adopted at a worldwide level to guarantee a homogeneous provision of care for chronic disease patients, considering also the geographical and cultural settings.

## An Innovative Approach to Improve the Future of RDs: RarERN Path™

As reported by the European pathways Association (EPA) “A care pathway is a complex intervention for the mutual decision making and organisation of care processes for a well-defined group of patients during a well-defined period^2^”. Therefore, considering that the main purpose of patients' care pathways (PCP) is to enhance the quality of care, their role is particularly crucial in the field of RDs and complex diseases as well. The context of the European Reference Networks (ERNs) might represent one of the most appropriate settings for the creation of organisational reference models for PCP across Europe. As a matter of fact, the main mission of ERNs is to improve the care of patients with RDs in Europe, through a patient-centred approach, thanks to real multistakeholder involvement.

For this reason, in the framework of the collaboration between the ERN on Rare and Complex Connective Tissue and Musculoskeletal Diseases ReCONNET (https://reconnet.ern-net.eu) Coordination Team and the group of Health Economics of the Institute of Management of the Scuola Superiore Sant'Anna, an extensive effort has been made towards the creation of a methodological approach aimed at providing organisational reference models for PCP in RDs across the different Member States. In fact, in order to develop the reference model, a structured methodology was created to enable the design of the PCP based on a deep sharing of expertise on high-quality care and characterised by a strong patient-centred approach: RarERN Path™ (7). RarERN Path™ represents a specific methodology aimed at improving organisation in real life and it was created by implementing the existing approaches already in use for the assessment of PCP with several innovative ways to look at the organisation itself. An organisation is, in fact, the core of the application of clinical practise guidelines (CPGs) or recommendations, and when an organisation fails it is really difficult to apply evidence-based guidelines in a homogeneous way. An *ad hoc* methodology was needed to address the specificity and the innovative asset provided by the ERNs and their unique environment represented by a multi-national and multi-stakeholder collaborative framework. RarERN Path™ brings the expertise of the different excellent centres across Europe at the local level, producing as the main result of its application, a reference organisational model that can be applied and adapted in a flexible way to different disease-specific and geographical contexts and that can be monitored and measured. As a matter of fact, an efficient implementation of existing clinical pathways can only be ensured by means of an efficient organisation of the healthcare systems and of the related services. Without an appropriate organisation, the journey of patients and of their caregivers can become long and exhausting and it can lead to limited and unequal access to care (8).

The RarERN Path™ approach foresees six consecutive phases, that start with mapping of what is in place in the different excellence centres, mainly focusing on the organisational aspects of the PCP, and collecting the perspectives of patients. This kind of approach provides the possibility to catch the different organisational challenges and best practises already in place and to design an optimised common PCP that reaches a consensus among the different stakeholders. Moreover, the co-design process with the different stakeholders is essential in the definition of disease-specific key performance indicators (KPI) able to monitor the application of the reference model and its advantages in terms of organisation and costs (7).

## Organisation as a Journey Towards Equity of Care

It is widely accepted that CPGs are defined as “statements that include recommendations intended to optimise patient care that is informed by a systematic review of evidence and an assessment of the benefits and harms of alternative care options” (9); however, one of the main barriers in the application of CPGs in daily care can not only be truly represented by local legislative restrictions, time constraints (10) but also by pragmatic difficulties in the organisation of PCP. Therefore, improving the organisational structure of PCP may surely contribute to a more efficient and sustainable application of the CPGs, especially in the case of different health contexts characterised by different expertise and resources.

Taking into account all these considerations, it becomes clear that improving the organisational aspects of PCP is particularly crucial in the field of RCs, where the knowledge is often scattered and access to care and treatment can be heterogeneous. Thus, improving organisation is definitely one of the main successful ways by which equity of care can be guaranteed across the different geographical areas. Improving organization of care means improving care to patients with RDs and it can be implemented in different ways; RarERN Path™ is definitely one of these ways. Thanks to the availability of appropriate methodologies aimed at improving the organisation of care, ERNs and other institutions can strongly commit to and support an improvement of the access both to treatments and to healthcare services for rare disease patients, thus contributing to increasing also more equity of care in RDs.

## The Added Value of Multi-Stakeholder Involvement in the Organisation of Care

**Figure 1:**
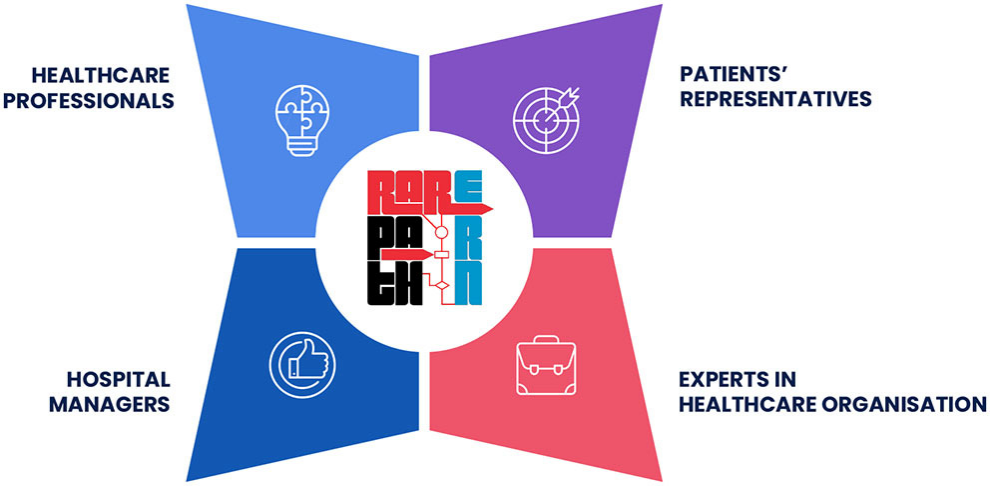
The different stakeholders involved in the application of RarERN Path™.

A multi-stakeholder approach ensures the identification and integration of the different needs and priorities in all contexts. Applying this approach in the organisation of care, for example in RarERN Path™, can be considered a must, as participatory processes ensure, among many factors, to define current challenges more accurately and to co-design strategies that are more tailored to the different needs. A tangible example of the RarERN Path™ multi-stakeholder involvement is, for example, the establishment of a patients' panel that is fully involved in the process, from the co-designing of the narrative medicine survey to the elaboration of the storeys and in the co-designing of the KPIs. Another example is represented by the consensus meeting (Phase 3 of RarERN Path™), in which expert clinicians gather with patients' representatives, economists, and hospital managers to discuss the patient's care pathway and the related organisation to be provided in order to deliver appropriate care to patients. Among the different stakeholders that need to be involved in planning strategic actions to ensure care also during an emergency, patients' representatives, healthcare professionals, hospital managers, and experts in a healthcare organisation play crucial roles (Figure 1). Their contribution should be encouraged and planned during all phases of the process in order to ensure on one side the applicability of the strategy designed and on the other side, that the needs and the priorities of all stakeholders are carefully addressed.

## Conclusion

Learning from the current pandemic is a necessary process that should ensure that vulnerable patients, such as patients with chronic and RD, will be able to access care also during health emergencies. A multi-stakeholder and multi-dimensional approach is needed to design health strategies that will enable RD PCP to continue to be active also in challenging conditions. RarERN Path™, represents, in this vision, a pragmatic approach that could be beneficial to the future of RD communities.

## Data Availability Statement

The original contributions presented in the study are included in the article/supplementary material, further inquiries can be directed to the corresponding author.

## Author Contributions

RT and DM written the paper. All authors discussed and agreed on the content, contributed on the message that the paper should bring to the readers, and approved the submitted version.

## Funding

This work was funded by the Call EU4H-2022-ERN-IBA-01 Project 101085769.

## Conflict of Interest

The authors declare that the research was conducted in the absence of any commercial or financial relationships that could be construed as a potential conflict of interest.

## Publisher's Note

All claims expressed in this article are solely those of the authors and do not necessarily represent those of their affiliated organizations, or those of the publisher, the editors and the reviewers. Any product that may be evaluated in this article, or claim that may be made by its manufacturer, is not guaranteed or endorsed by the publisher.

## Footnotes

^1^ https://covid19.who.int/

^2^ http://e-p-a.org/
